# Ischemia during rest intervals between sets prevents decreases in fatigue during the explosive squat exercise: a randomized, crossover study

**DOI:** 10.1038/s41598-022-10022-4

**Published:** 2022-04-08

**Authors:** Robert Trybulski, Jakub Jarosz, Michal Krzysztofik, Milena Lachowicz, Grzegorz Trybek, Adam Zajac, Michal Wilk

**Affiliations:** 1 Provita Zory Medical Center, 44-240 Zory, Poland; 2 Department of Medical Sciences, The Wojciech Korfanty School of Economics, 40-065 Katowice, Poland; 3 Institute of Sport Sciences, Jerzy Kukuczka Academy of Physical Education in Katowice, ul Mikołowska 72a, 40-065 Katowice, Poland; 4 Faculty of Physical Education, Gdansk University of Physical Education and Sport, 80-336 Gdansk, Poland; 5 Department of Oral Surgery, Pomeranian Medical University, 72 Powstanców Wlkp. St., Szczecin, Poland

**Keywords:** Skeletal muscle, Quality of life

## Abstract

The study aimed to evaluate the impact of ischemia, used only before particular sets of a lower limb resistance exercise on power output. Ten healthy resistance-trained males (age = 26 ± 6 years; body mass = 90 ± 9 kg; training experience = 9 ± 7 years) performed two experimental sessions (with ischemia; control without ischemia) following a randomized crossover design. During the ischemic condition, the cuffs were inflated to 60% of arterial occlusion pressure. The cuffs were applied before each set for 4.5 min and released 30 s before the start of the set as the reperfusion (4.5 min ischemia + 0.5 min reperfusion). In the control condition, ischemia was not applied. During the experimental sessions, the subjects performed the Keiser machine squat exercise protocol which consisted of 5 sets of two repetitions, at a load of 60% of one-repetition maximum (1RM), with 5 min rest intervals between sets. The repetitions were performed with maximal velocity. The two-way repeated-measures ANOVA showed a statistically significant interaction effect for power output (*p* < 0.01; η^2^ = 0.26). There was also a statistically significant main effect of condition for power output (*p* = 0.02; η^2^ = 0.40). The post hoc analysis for interaction did not show significant differences between conditions in particular sets. The post hoc analysis for the main effect of the condition revealed that power output was significantly lower in the control group compared to the group where ischemic was used (*p* = 0.02). The t-test comparisons for particular sets showed a significant lower power output in set 3 (*p* = 0.03); set 4 (*p* < 0.01) and set 5 (*p* < 0.01) for the control condition when compared to the ischemic condition. The results indicate that ischemia applied before each set and released 30 s prior to the start of the squat exercise did not increase power output performance. However, we observed a significantly lower decline in power for the ischemic condition (4.5 min ischemia + 0.5 min reperfusion) in sets 3–5 compared to the control condition. Thus repeated ischemia with reperfusion used between sets can be an effective form of performance enhancement by preventing or at least diminishing fatigue during resistance exercise.

## Introduction

Ischemia used between sets or intra-conditioning is a method of blocking blood flow for a specified duration (usually from a few to several minutes) and then releasing it, causing blood reperfusion before performing a physical exercise^1^. Ischemia is simple, non-invasive, affordable, and, thereby, easy to apply before, between, or during physical exercise^2^. Furthermore, there are different methods of using ischemia, such as continuous (applied during exercise and rest periods), intermittent (applied only during exercise), or pre-conditioning ischemia (used only before exercise). The tissues, previously submitted to blood flow restriction, become more resistant to ischemia arising during exercise and its potential deleterious effects^3^. Previous studies demonstrated the beneficial effects of ischemic pre-conditioning on performance during swimming, running, cycling, and resistance exercise^4–8^. While ischemia can improve exercise performance and stimulate several physiological responses^7,9–15^ the mechanisms underlying its effects are unclear.

Despite that much attention has been focused on ischemia used before physical exercise^4–8^, only one study has considered the effect of ischemia applied during rest intervals between successive sets of a resistance exercise on power output. Wilk et al.^16^ showed that the ischemia used during rest intervals significantly increase power output and bar velocity during the bench press exercise at 60% 1 RM (5 sets of 3 repetitions). Therefore, the results of this study indicate that ischemic intra-conditioning can effectively improve upper body performance during a multi-set resistance exercise^16^. It is interesting to note that the increase of performance for the ischemic condition observed in the study of Wilk et al.^16^ particularly in sets 3–5, suggests that ischemia used before sets allows the athletes to maintain a certain amount of power output even in the presence of biochemical changes within the working muscles that lead to fatigue. The increase in power output was also observed when ischemic conditioning was applied during exercise. The study by Wilk et al.^17^ showed that intermittent (used only during exercise), high pressure ischemic arterial occlusion pressure (AOP) with a wide cuff, increases bar velocity and power output during the bench press exercise at 70% 1 RM. Further, Wilk et al.^18^ showed that ischemic pressure (70% AOP), continuous as well as intermittent with a narrow cuff can increase bar velocity during the bench press exercise at loads of 20–50% 1 RM but not at higher loads. However, when similar test procedures as those mentioned above were used for lower limb exercise the results were inconsistent and did not show a significant increase in power output following ischemic treatment^19^. Therefore, the acute effect of ischemic pressure on power output during resistance exercise is related to the area of muscle where ischemia is applied and its effect may be different in the upper and lower limbs. However, there is currently no scientific data assessing the effects of ischemia applied only during rest intervals between successive sets of lower limb resistance exercise.

Since the squat exercise is a basic resistance exercise for developing lower body strength and power^20–23^, the aim of the study was to evaluate the influence of ischemic pressure, used only during rest intervals between successive sets of the squat exercise on power output. It was hypothesized that ischemia would increase power output during the squat exercise.

## Materials and methods

**Figure 1:**
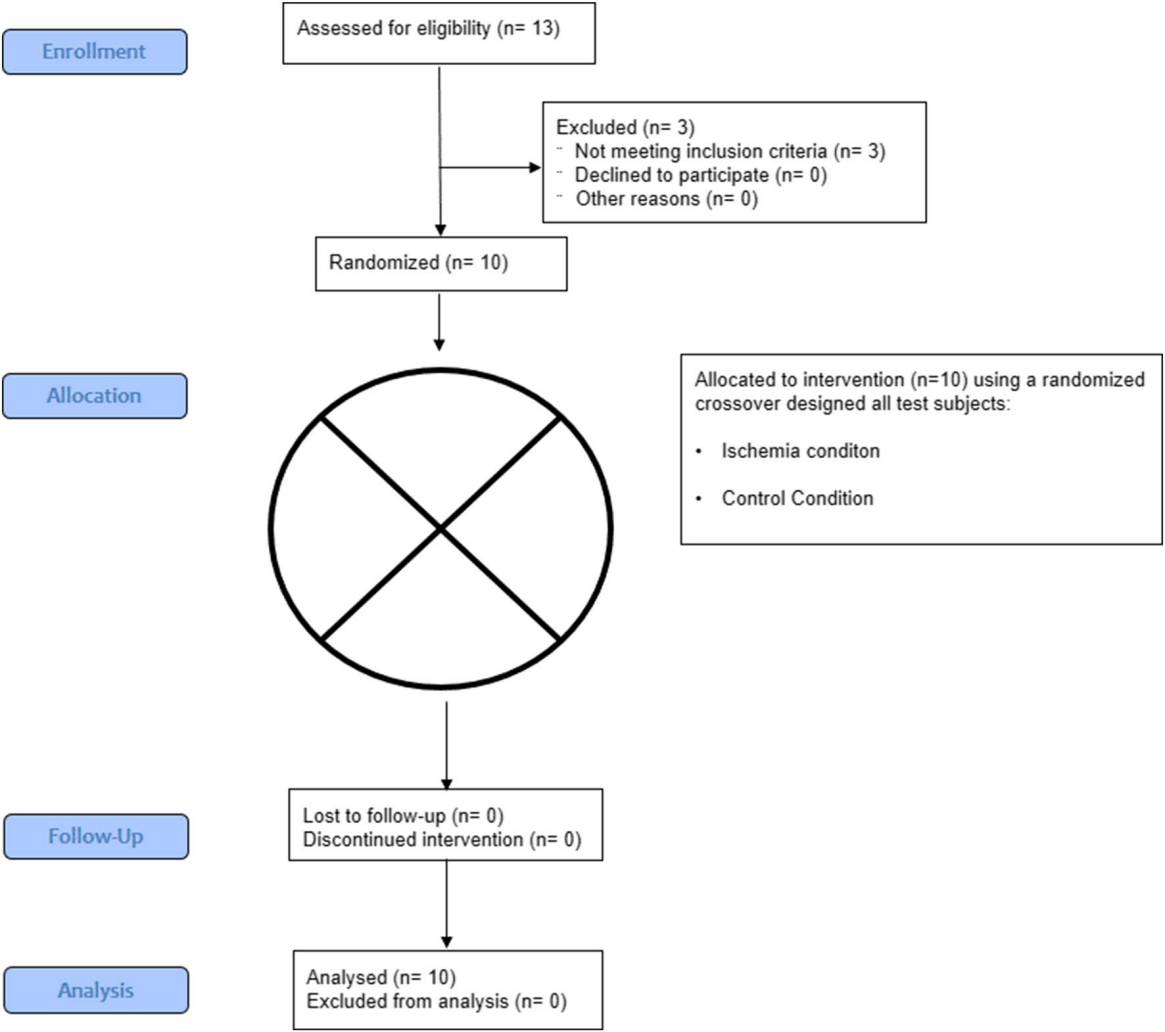
CONSORT flow diagram.

The experiment was performed following a randomized crossover design, where each subject performed two different testing protocols in random and counterbalanced order, one week apart: without ischemia (control condition) and with ischemic pressure used before exercise and during the rest intervals between sets (ischemic conditions, Fig. 1). During each experimental session, the participants performed 5 sets of 2 repetitions of the squat with the Keiser Air-300 Squat Machine (Keiser, Fresno, CA, USA) with a load of 60% 1 RM, and a 5 min rest interval between each set. The external load was chosen based upon the range indicated as optimal for obtaining peak power outputs during a squat exercise^24^. The peak power output was a pre-specified primary outcome measure. There were no secondary outcome measures. Peak power output was evaluated by the Keiser Air-300 Squat Machine which is a pneumatic resistance system that utilizes air-pressurized resistance to maximize safety and allows for a precise adjustment of external load. All testing sessions were performed in the Strength and Power Laboratory at the Academy of Physical Education in Katowice, Poland. The manuscript was prepared according to the CONSORT guidelines. The experimental procedure has not changed at any stage of the experiment. No interim analyses were performed, the trial had no stopping guidelines. Further, there were no changes to trial outcomes after the trial commenced.

### Subjects

Ten healthy resistance-trained males volunteered for the study after completing an informed consent form (age = 26 ± 6 years; body mass = 90 ± 9 kg; Keiser machine squat 6RM = 208 ± 34 kg; training experience = 9 ± 7 years). The inclusion criteria were as follows: (1) free from neuromuscular and musculoskeletal disorders, (2) not to use any supplements or stimulants for the duration of the experiment. The participants were informed about the main goal of the study and about the potential risks before providing their written informed consent for participation. The participants were allowed to withdraw from the study at any time. To calculate the sample size, statistical software (G*Power, Dusseldorf, Germany) was used. Given the study 2-way analysis of variance (ANOVA) (2 conditions and 5 repeated measures), a medium overall effect size (ES) = 0.5, an alpha-error < 0.05, and the desired power (1 − β error) = 0.8, the total sample size resulted in 9 participants. The experimental protocol was approved by the Bioethics Committee, at the Academy of Physical Education in Katowice, Poland (02/2019).

### Procedures

#### Familiarization session

Two weeks before the experimental sessions, the participants performed one familiarization session. During the familiarization session, each participant performed a standardized warm-up that was consistent with the subject’s normal training habits and next performed 5 sets of 2 repetitions of the Keiser machine squat with ischemic pressure used before and during the rest intervals at load 60% 6 RM. In the familiarization session, the lowest position of the movement was found—defined as when the trochanter major aligned with the upper part of the patella. The familiarization session was performed in order to minimize possible learning effects during the main tests.

#### Six repetitions maximum test (6 RM)

One week before the main experiment the 6 RM Keiser machine squat test was performed. In the 6RM test, the load started from the load achieved in the familiarization test. The load was either increased or decreased by 2.5–10 kg until 6 RM was reached. All subjects reached their 6 RM loads within 5 attempts. The testing was terminated if the subjects were not able to complete all 6 repetitions of a set if the subject and the test leader agreed that the subject could not lift a higher weight, or if the subjects were not able to maintain proper technique. A 4–5 min rest interval was given between each attempt.

### Experimental sessions

In a randomized and counterbalanced order, the subjects performed the squat exercise on the Keiser Air-300 Squat Machine under 2 different testing conditions: without ischemia (control condition); with ischemia (ischemic condition) used before exercise and during the rest periods between sets. The research team was blinded. The randomization was performed by a blinded member of the research team who was not involved in the data collection. The person carrying out the measurements did not participate in the data collection. The person performing the statistical analysis was not involved in other stages of the experiment. The order of trials (Ischemia, control) was chosen randomly and in a counterbalanced order using a free online randomization program (randomization.com). During each experimental session, the participant performed 5 sets of the Keiser machine squat at a load of 60% 1 RM with a 5 min rest period between sets. During each set, the participants performed 2 reps, with a maximal tempo of movement^25,26^. A Keiser Air-300 Squat Machine was used for the evaluation of peak power output. Measurements were made independently for each repetition. The power output was obtained from the best repetition performed in a particular set. Based on measurements from the familiarization session, the intra-class correlation coefficient for the test–retest reliability for peak power output during the Keiser Air 300 Squat was 0.94. All subjects completed the described experimental protocol that was carefully replicated in subsequent experimental sessions.

### Ischemic procedure

During the ischemic condition, the participants applied pressure cuffs at the most proximal region of both lower limbs above the foot (on the thigh). For this experiment, Smart Cuffs were used (Smart Tools Plus LLC, Strongsville, USA). To determine individual pressure, after a 5 min rest interval, the value of full arterial occlusion pressure was determined^17,27^. The cuff pressure for the Keiser machine squat exercise was set to ~ 60% of full arterial occlusion pressure (183 ± 17 mmHg). The level of vascular restriction was monitored using a handheld Edan SD3 Doppler with an OLED screen and a 2 MHz probe produced by Edan Instruments (Shenzen, China)^17,27^. For the ischemic condition, the occlusion was applied before each set for 4.5 min and released 30 s upon the start of the set as the reperfusion (4.5 min ischemia + 0.5 min reperfusion).

### Statistical analysis

All statistical analyses were performed using Statistica 9.1. Results are presented as means with standard deviations. There was no similarity of interventions. The Shapiro–Wilk test was performed in order to verify the normality, homogeneity and sphericity of the sample data variances. The significance of differences (*p* < 0.05) between the ischemia and control conditions were examined using repeated measures 2-way analysis of variance ANOVA (2 conditions × 5 sets). Effect sizes for interactions and the main effects were analyzed by partial eta squared (η^2^; small = 0.01 to 0.059; moderate = 0.06–0.137; large > 0.137). Post hoc comparisons using Tukey’s test were conducted to locate the differences. Furthermore, paired t-test comparisons between conditions were made for particular sets. To pairwise comparisons, Cohen’s d effect sizes were performed (large *d* > 0.8; moderate *d* between 0.8 and 0.5; small *d* between 0.49 and 0.20; trivial *d* < 0.2) (Cohen, 1988). Percent changes with 95% confidence intervals (95 CI) were also calculated.

## Results

The two-way repeated measures ANOVA showed a statistically significant interaction effect for power output (*p* < 0.01; η^2^ = 0.26). There was also statistically significant main effect of condition for power output (*p* = 0.02; η^2^ = 0.40).

The post hoc analysis for interaction did not show significant differences between conditions in particular sets. The post hoc analysis for the main effect of the condition revealed that power output was significantly higher for the ischemic compared to the control condition (*p* = 0.02; Table 1).

**Table 1:** Power output during five sets of the Keiser machine squats following ischemic pressure and control conditions.

Condition	Set 1 [w] (95% CI)	Set 2 [w] (95% CI)	Set 3 [w] (95% CI)	Set 4 [w] (95% CI)	Set 5 [w] (95% CI)	*p* for interaction	*p* for main effect
Control	2214 ± 637 (1809–2619)	2234 ± 653 (1820–2649)	2172 ± 646 (1761–2582)	2153 ± 609 (1766–2539)	2172 ± 610 (1784–2559)	< 0.01	0.02
Ischemic	2223 ± 626 (1826–2621)	2184 ± 603 (1801–2568)	2242 ± 627 (1844–2641)	2219 ± 619 (1826–2612)	2237 ± 641 (1830–2645)		
*p* for *t*-test	0.84	0.02	0.03	< 0.01	< 0.01		
Effect size	0.01	0.08	0.11	0.11	0.10		

All data are resented as mean SD; CI = confidence interval; statistically significant differences *p* < 0.05.

The *t* test comparisons for particular sets showed a significant lower power output in set 3 (*p* = 0.03); set 4 (*p* < 0.01) and set 5 (*p* < 0.01) for the control condition when compared to the ischemic condition (Table 1). Furthermore, the t-test showed a significant decrease in power output in set 2 (*p* = 0.02) for the ischemic treatment when compared to control conditions (Table 1). There were no performed demographic and clinical characteristics for a group as well as there was no important harms or unintended effects.

## Discussion

The main finding of the presented study was that ischemic pressure applied during the rest intervals between sets does not increase power output during a Keiser squat training session at 60% 1 RM. However, it was observed that ischemia during the rest period between sets and released 30 s prior to the start of the test prevented progressive fatigue and thus the decrease in power output in subsequent sets compared to the control condition. The analysis performed independently for each set showed significantly lower power output in the control condition in sets 3–5 when compared to the ischemia condition. Therefore, these results indicate that ischemia applied during the rest interval between successive sets and released 30 s prior to the start of the next set does not increase power output, but can be effective in maintaining performance during multi-set resistance training sessions.

To the best of the author's knowledge, only one previous study assessed the effects of ischemic pressure applied during rest intervals between sets of resistance exercise on power output^16^. Wilk et al.^16^ showed that ischemia applied during the rest periods between resistance exercises significantly increase power output and bar velocity of the upper limbs (bench press exercise; 60% 1 RM; 5 sets of 3 repetitions), however, the presented study showed that ischemic pressure applied during rest intervals between sets reduced decreases in power output in subsequent sets, yet this intervention did not increase peak power output. However, in the present study, ischemia was applied to the lower limbs, while in the Wilk et al.^16^, study, the ischemic treatment was applied to the upper limbs. Therefore, the results of both studies confirmed that ischemia applied during rest intervals can be an effective tool for power output performance during resistance exercise, regardless of the muscle area where the tourniquet was applied. In the presented study the ischemic treatment prevented the arising fatigue but did not increase explosive performance, which is contrary to the study of Wilk et al.^16^. However considering the power output during the entire multi-set resistance training session, the applied ischemia can be interpreted as a benefit and the desired response. The minor decreases in power output for the ischemic condition were observed particularly in sets 3–5, which suggests that this type of intervention during the rest intervals allows the athletes to maintain power output even in the presence of biochemical changes within the working muscles that lead to fatigue^16^. The muscle previously submitted to ischemic pressure becomes more resistant to fatigue during subsequent resistance exercise and power output decreases to a smaller extent in successive sets^3^.

Therefore the use of ischemia not only during pre-conditioning warm-up^4–8^ but also repeated ischemic treatment between sets may enhance physical performance^16^. The observed maintenance of power output in the ischemic condition in subsequent sets (3–5), may confirm that the effectiveness of ischemia is observed in later stages of a resistance exercise performed under significant fatigue. In set 1 no significant difference between conditions was observed, while in set 2 a significantly lower power output for the ischemic condition was observed compared to the control, which indicates that at least three ischemic interval treatments have to be applied to enhance performance.

The maintenance of power performance due to ischemia can be justified by physiological factors similar to those observed for ischemic pre-conditioning. Ischemia causes positive physiological changes in absorption kinetics of pulmonary O_2_, in the systemic VO_2_, in the deoxygenation of muscular Hb/Mb, and the opening of the ATP-dependent K^+^ channels by increasing the energy stocks after ischemia and in muscle vasodilation^9–12,28–31^. Furthermore, Torma et al.^32^ reported that ischemia, applied only during rest intervals between sets, may impact the gene expression of angiogenesis, mitochondrial biogenesis, affecting the time of muscle repair, and hypertrophy responses associated with increased protein synthesis in cultured myotubes^33^. Therefore the decreases miR-206 levels and increases expression of Pax7 following ischemia may affect the adaptive response to resistance exercise^32,33^. Therefore, ischemic treatments used during rest intervals may not only impact acute physical performance but may also be a factor in stimulating chronic adaptations following resistance exercise.

The positive effect of ischemia on power performance during multi-set exercise can also be related to the more efficient use of post-activation performance enhancement (PAPE)^16,34^. PAPE is a muscle phenomenon that allows a short-term increase of power output production due to a prior muscle activation^35–39^. Some studies have shown a beneficial effect of previous muscle activation on power output generated in consecutive sets of resistance exercises^34,39^. Therefore, the maintenance of power output in the ischemic condition can be related to the more effective use of the PAPE effect, which was previously suggested by Wilk et al.^16^. Therefore, similar to the PAPE effect it seems that not only the repeated use of ischemia but also the ratio of exercise duration (time under tension) to the duration of ischemic treatment during the rest interval can affect power performance^39–41^. Consequently, the number of performed sets, repetitions, and the ratio of effort to the duration of ischemia and the duration of the rest interval, can significantly affect power output following such a treatment. During the presented research protocol the participants performed a low number of repetitions for each set and such short efforts are fueled mainly by phosphocreatine and anaerobic glycolysis, which are largely restored during the rest interval^42,43^. The effectiveness of ischemia used during the rest intervals of resistance exercises performed to failure, my change. However, few previous studies have shown an increase in performance following ischemic pre-conditioning, even when the effort was performed to failure^8,44,45^. Telles et al.^44^ showed that the maximal number of repetitions in the bench press and leg press exercise was higher after an ischemic pre-conditioning protocol compared to control conditions^8,45^. The total volume of work was also higher following ischemic pre-conditioning compared to other warm-up testing protocols^44^. However, currently there are no studies that have assessed the effect of ischemia used during rest periods on strength-endurance performance, which requires further research into the topic.

The results of this study show that ischemia used during rest intervals between sets of resistance exercise may be beneficial for power performance. However, there are some limitations of the study that need to be addressed. There were no assessments of physiological markers that could provide a possible explanation for maintenance power output for the ischemic condition. Another limitation is the lack of a placebo group in the experimental procedure. The placebo and/or nocebo effects are both methodological confounding factors in studies involving potential ergogenic aids^4,6,46,47^.

## Conclusions

The result of the present study, suggests that ischemia used during rest intervals between sets can be an effective tool to prevent significant decreases in power output during explosive Keiser squat exercise. Thus, it can be speculated that using repetitive ischemic treatment between sets of the Keiser squats can be an effective form of performance enhancement and may diminish fatigue in other resistance exercises, as well as in specific explosive movements such as jumps, throws, or sprints. It should be noted that the application of repeated ischemia may induce acute post-exercise responses, as well as chronic adaptive changes by increasing the expression of genes for angiogenesis, mitochondrial biogenesis, during the time of muscle repair. Therefore, ischemia used during rest intervals not only enhances acute kinematic performance but may also be a factor in stimulating chronic adaptive changes related to muscular strength and hypertrophy. However, in order for the ischemic treatment to induce positive changes in power performance, it should be applied several times during rest intervals in a single training session.

## References

[1] Eltzschig HK, Eckle T (2011). Ischemic and reperfusion—from mechanism to translation. Nat. Med..

[2] Marocolo M, Billaut F, da Mota GR (2018). Ischemic preconditioning and exercise performance: An ergogenic aid for whom?. Front. Physiol..

[3] Kocman EA (2015). Effects of ischemic preconditioning protocols on skeletal muscle ischemic–reperfusion injury. J. Surg. Res..

[4] Marocolo M, Da Mota GR, Pelegrini V, Appell Coriolano HJ (2015). Are the beneficial effects of ischemic preconditioning on performance partly a placebo effect?. Int. J. Sports Med..

[5] Ferreira TN, Sabino-Carvalho JL, Lopes TR (2016). Ischemic preconditioning and repeated sprint swimming: A placebo and nocebo study. Med. Sci. Sports. Exerc..

[6] Sabino-Carvalho JL (2017). Effect of ischemic preconditioning on endurance performance does not surpass placebo. Med. Sci. Sports Exerc..

[7] Paradis-Deschênes P, Joanisse DR, Billaut F (2016). Ischemic preconditioning increases muscle perfusion, oxygen uptake, and force in strength-trained athletes. Appl. Physiol. Nutr. Metab..

[8] Marocolo M (2016). Beneficial effects of ischemic preconditioning in resistance exercise fade over time. Int. J. Sports Med..

[9] Lawson C, Downey J (1993). Preconditioning: State of the art myocardial protection. Cardiovasc. Res..

[10] Pang CY (1995). Acute ischaemic preconditioning protects against skeletal muscle infarction in the pig. Cardiovasc. Res..

[11] Li XD (2012). PKA-mediated eNOS phosphorylation in the protection of ischemic preconditioning against no-reflow. Microvasc. Res..

[12] Kimura M (2007). Repetition of ischemic preconditioning augments endothelium-dependent vasodilation in humans: Role of endothelium-derived nitric oxide and endothelial progenitor cells. Arterioscler. Thromb. Vasc. Biol..

[13] Liu GS (1991). Protection against infarction afforded by preconditioning is mediated by A1 adenosine receptors in rabbit heart. Circulation.

[14] Schroeder CA (1996). Preconditioning with ischemic or adenosine protects skeletal muscle from ischemic tissue reperfusion injury. J. Surg. Res..

[15] Tanaka D (2018). Ischemic preconditioning enhances muscle endurance during sustained isometric exercise. Int. J. Sports Med..

[16] Wilk, M. et al. Impact of ischemic intra-conditioning on power output and bar velocity of the upper limbs. *Front. Physiol.*10.3389/fphys.2021.626915 (2021) [Epub ahead of print]. 10.3389/fphys.2021.626915 PMC7947627 33716773

[17] Wilk, M. et al. Short-term blood flow restriction increases power output and bar velocity during the bench press. *J. Strength Cond. Res*. 10.1519/JSC.0000000000003649 (2020) [Online ahead of print]. 10.1519/JSC.0000000000003649 32379236

[18] Wilk M (2020). Acute Effects of continuous and intermittent blood flow restriction on movement velocity during bench press exercise against different loads. Front. Physiol..

[19] Gepfert M, Golas A, Zajac T, Krzysztofik M (2020). The use of different modes of post-activation potentiation (PAP) for enhancing speed of the slide-step in basketball players. Int. J. Environ. Res..

[20] Myer GD (2014). The back squat: A proposed assessment of functional deficits and technical factors that limit performance. Strength Cond. J..

[21] Wilk M (2018). Endocrine responses following exhaustive strength exercise with and without the use of protein and protein–carbohydrate supplements. Biol. Sport..

[22] Wilk M, Petr M, Krzysztofik M, Zajac A, Stastny P (2018). Endocrine response to high intensity barbell squats performed with constant movement tempo and variable training volume. Neuro Endocrinol. Lett..

[23] Lee JY, Lee DY (2018). Effect of different speeds and ground environment of squat exercises on lower limb muscle activation and balance ability. Technol. Health Care..

[24] Siegel JA, Gilders RM, Staron RS, Hagerman FC (2002). Human muscle power output during upper- and lower-body exercises. J. Strength Cond. Res..

[25] Wilk M (2020). Impact of duration of eccentric movement in the one-repetition maximum test result in the bench press among women. J. Sports Sci. Med..

[26] Wilk M (2020). The effects of the movement tempo on the one-repetition maximum bench press results. J. Hum. Kinet..

[27] Wilk M, Krzysztofik M, Filip A, Lockie RG, Zajac A (2020). The acute effects of external compression with blood flow restriction on maximal strength and strength-endurance performance of the upper limbs. Front. Physiol..

[28] Paganelli W, Pendergast DR, Koness J, Cerretelli P (1989). The effect of decreased muscle energy stores on the VO_2_ kinetics at the onset of exercise. Eur. J. Appl. Physiol. Occup. Physiol..

[29] de Groot PC, Thijssen DH, Sanchez M, Ellenkamp R, Hopman MT (2010). Ischemic preconditioning improves maximal performance in humans. Eur. J. Appl. Physiol..

[30] Barbosa TC (2015). Remote ischemic preconditioning delays fatigue development during handgrip exercise. Scand. J. Med. Sci. Sports..

[31] Paradis-Deschênes P, Joanisse DR, Billaut F (2016). Ischemic preconditioning increases muscle perfusion, oxygen uptake, and force in strength-trained athletes. Appl. Physiol. Nutr. Metab..

[32] Torma F (2019). Blood flow restriction in human skeletal muscle during rest periods after high-load resistance training down-regulates miR 206 and induces Pax7. J. Sport Health. Sci..

[33] Winbanks CE (2013). MiR-206 represses hypertrophy of myogenic cells but not muscle fibersvia inhibition of HDAC4. PLoS ONE.

[34] Wilk M (2020). Does post-activation performance enhancement occur during the bench press exercise under blood flow restriction?. Int. J. Environ. Res. Public. Health.

[35] Krzysztofik, M., Wilk, M., Stastny, P. & Golas, A. Post-activation performance enhancement in the bench press throw: A systematic review and meta-analysis. *Front. Physiol*. **11**, 598628 (2021). 10.3389/fphys.2020.598628 PMC7844331 33519506

[36] Krzysztofik M, Wilk M (2020). The effects of plyometric conditioning on post-activation bench press performance. J. Hum. Kinet..

[37] Krzysztofik M (2020). Does Eccentric-only and concentric-only zctivation increase power output?. Med. Sci. Sports Exerc..

[38] Krzysztofik, M. et al. Postactivation performance enhancement of concentric bench press throw after eccentric-only conditioning exercise. *J. Strength Cond. Res*. **10**, 1519. 10.1519/JSC.0000000000003802 (2020). 10.1519/JSC.0000000000003802 32826834

[39] Wilk M, Krzysztofik M, Drozd M, Zajac A (2020). Changes of power output and velocity during successive sets of the bench press with different duration of eccentric movement. Int. J. Sports Physiol. Perform..

[40] Chaouachi A (2011). Volume, intensity, and timing of muscle power potentiation are variable. Appl. Physiol. Nutr. Metab..

[41] Gossen ER, Sale DG (2000). Effect of postactivation potentiation on dynamic knee extension performance. Eur. J. Appl. Physiol..

[42] Bogdanis GC, Nevill ME, Lakomy HKA, Boobis LH (1998). Power output and muscle metabolism during and following recovery from 10 and 20 s of maximal sprint exercise in humans. Acta Physiol. Scand..

[43] Dawson B (2007). Muscle phosphocreatine repletion following single and repeated short sprint efforts. Scand. J. Med. Sci. Sports..

[44] da Silva Telles, L. G. et al. Effects of ischemic preconditioning as a warm-up on leg press and bench press performance. *J. Hum. Kinet.* **31**, 267–277 (2020). 10.2478/hukin-2020-0055 PMC7706675 33312313

[45] Marocolo M (2016). Ischemic preconditioning and placebo intervention improves resistance exercise performance. J. Strength Cond. Res..

[46] Marocolo IC (2017). Acute ischemic preconditioning does not influence high-intensity intermittent exercise performance. Peer J..

[47] Wilk M, Krzysztofik M, Maszczyk A, Chycki J, Zajac A (2019). The acute effects of caffeine intake on time under tension and power generated during the bench press movement. J. Int. Soc. Sports Nutr..

